# In silico discovery of substituted pyrido[2,3-*d*]pyrimidines and pentamidine-like compounds with biological activity in myotonic dystrophy models

**DOI:** 10.1371/journal.pone.0178931

**Published:** 2017-06-05

**Authors:** Àlex L. González, Piotr Konieczny, Beatriz Llamusi, Estefanía Delgado-Pinar, José I. Borrell, Jordi Teixidó, Enrique García-España, Manuel Pérez-Alonso, Roger Estrada-Tejedor, Rubén Artero

**Affiliations:** 1 Grup d’Enginyeria Molecular (GEM), Institut Químic de Sarrià (IQS)–Universitat Ramon Llull (URL), Barcelona, Catalonia, Spain; 2 Translational Genomics Group, Incliva Health Research Institute, Valencia, Spain; 3 Department of Genetics and Interdisciplinary Research Structure for Biotechnology and Biomedicine (ERI BIOTECMED), University of Valencia, Valencia, Spain; 4 Incliva-CIPF joint unit, Valencia, Spain; 5 Instituto de Ciencia Molecular, Universidad de Valencia, Paterna, Valencia, Spain; University of Parma, ITALY

## Abstract

Myotonic dystrophy type 1 (DM1) is a rare multisystemic disorder associated with an expansion of CUG repeats in mutant *DMPK* (dystrophia myotonica protein kinase) transcripts; the main effect of these expansions is the induction of pre-mRNA splicing defects by sequestering muscleblind-like family proteins (e.g. MBNL1). Disruption of the CUG repeats and the MBNL1 protein complex has been established as the best therapeutic approach for DM1, hence two main strategies have been proposed: targeted degradation of mutant *DMPK* transcripts and the development of CUG-binding molecules that prevent MBNL1 sequestration. Herein, suitable CUG-binding small molecules were selected using in silico approaches such as scaffold analysis, similarity searching, and druggability analysis. We used polarization assays to confirm the CUG repeat binding in vitro for a number of candidate compounds, and went on to evaluate the biological activity of the two with the strongest affinity for CUG repeats (which we refer to as compounds **1**–**2** and **2**–**5**) in DM1 mutant cells and Drosophila DM1 models with an impaired locomotion phenotype. In particular, **1**–**2** and **2**–**5** enhanced the levels of free MBNL1 in patient-derived myoblasts in vitro and greatly improved DM1 fly locomotion in climbing assays. This work provides new computational approaches for rational large-scale virtual screens of molecules that selectively recognize CUG structures. Moreover, it contributes valuable knowledge regarding two compounds with desirable biological activity in DM1 models.

## Introduction

Myotonic dystrophy type 1 (DM1) originates from a progressive expansion of CTG repeats in the 3’-unstranslated region of the dystrophia myotonica protein kinase (*DMPK*) gene [1,2]. Once transcribed, CUG expanded-repeats (abbreviated rCUG^exp^) fold into long stable RNA hairpins and accumulate in nuclear structures called ribonuclear foci [3,4]. The high affinity that all three human muscleblind-like (MBNL) protein paralogues have for rCUG^exp^ structures results in their sequestration and depletion from normal cellular targets. Thus, DM1 was the first disease with a described RNA-gain-of-function pathogenic mechanism [5]. MBNL1 is a key regulator of muscle, heart, and brain transcript alternative-splicing, including in some genes that have been associated with specific DM1 symptoms such as insulin receptor (*IR*) [6], cardiac troponin T (*cTNT*) [7], and muscle-specific chloride ion channel (*CLCN1*) [8].

The rCUG^exp^ structure has been successfully targeted to prevent MBNL depletion by using several different approaches, including the use of *D*-amino acid hexapeptides [9], antisense oligonucleotides [10], or small molecules [11–15]. The potential for targeting RNA using small molecules is overwhelming and several scaffolds have already been proven to reverse MBNL1 sequestering (e.g. bis-benzimidazoles) [11–16]. In an effort to explore the chemical space available, Chen et al. performed a quantitative high-throughput screening (qHTS) of more than 300,000 compounds to identify those able to inhibit the formation of r(CUG)_12_–MBNL1 complexes [13]. In vitro testing of some of these compounds showed that they had distinct molecular mechanisms and divergent results in the missplicing rescue. Among these, lomofungin and its dimer, dilomofungin, were identified as potential inhibitors due to the potency of their rCUG^exp^ binding. However, unexpected effects on RNA decay were reported, suggesting that they have an alternative, undesirable mechanism of action in vitro [14].

Zimmerman and co-workers followed a multivalent ligand strategy to cooperatively bind rCUG^exp^, based on previously reported triaminotriazine-acridine conjugates which have a high nanomolar affinity to them [17,18]. The bivalent ligand exhibited greater inhibition potency than the monomer in a DM1 cell model, however, the inherent cytotoxicity of intercalators led to the design of groove-binding ligands carrying two triaminotriazine units [18]. In that study, Wong et al. reported partial relief of MBNL1 sequestration and was able to reverse the eye phenotype in a DM1 Drosophila model [18].

The identification of pentamidine and Hoechst 33258 as privileged scaffolds for disrupting rCUG^exp^–MBNL1 interactions led to the discovery of a new set of compounds with a similar shape and chemical features [19,20]. Using chemical similarity searching, Parkesh et al. identified a Hoechst derivative that improved DM1-associated splicing defects in cellular and animal models [16]. Furthermore, computer-aided molecular design may boost the identification of new drugs by applying structure-based drug-design techniques that allow the formation of the RNA-ligand complex and its dynamic behavior to be to simulated [21–22]. However, the lack of NMR and X-ray structural models of RNA-small molecule complexes hampers the ability of in silico approaches to validate intensive virtual screening protocols.

Without minimizing the importance of the classical lock-and-key or complementary-shaped bodies model, it is increasingly becoming clear that RNA molecular recognition requires the flexibility of macromolecules and their potential structural changes to be considered [23,24]. According to the conformational selection model postulated by Monod, Wyman and Changeux, different macromolecule conformations exist in dynamic equilibrium prior to ligand binding. This binding would shift the equilibrium to a bound state by recognizing and stabilizing one such conformation [25]. Following this principle, herein we describe a combined ligand and structure-based drug design approach that led to the discovery of a novel class of inhibitor scaffold with promising activity against DM1. We combined essential dynamics analysis (EDA), which predicts intrinsic RNA dynamics using experimentally resolved rCUG^exp^ structures, and molecular docking, whose ability to predict bound conformations has been extensively probed. Importantly, this study provides a fast and rational approach for designing new chemical entities which recognize rCUG^exp^ structures.

## Results

### Classification of compounds based on radial fingerprints

**Fig 1:**
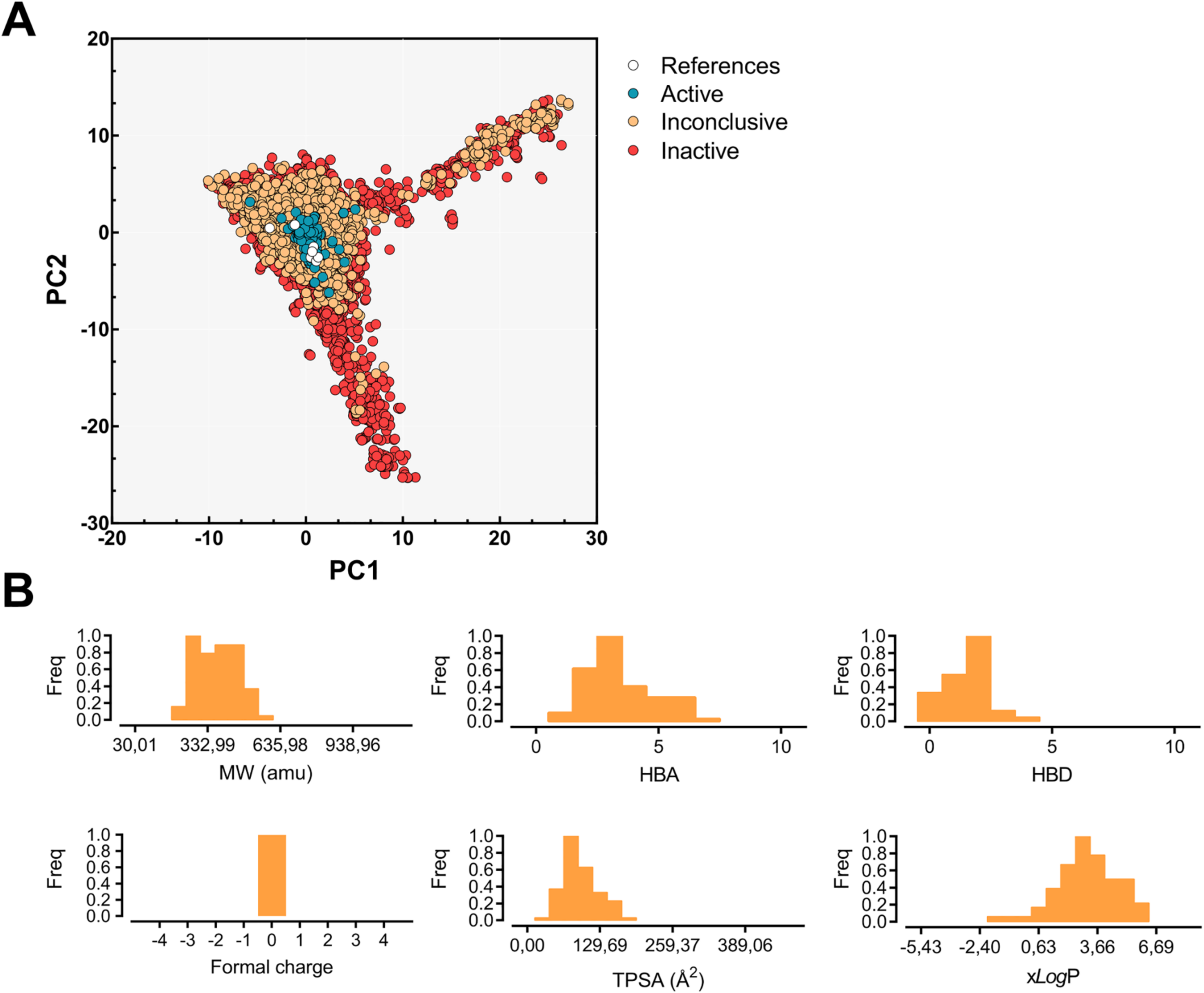
(A) Radial fingerprint projection over the PC1 and PC2 subspace. Inactive (red), inconclusive (orange), and active molecules (blue) are scattered over the subspace. White dots represent previously reported bioactive molecules [13,14,26]. (B) The observed frequencies of the main physicochemical property distribution characteristics in the chemical dataset: molecular weight (MW), hydrogen bond acceptors (HBAs), hydrogen bond donors (HBDs), formal charge, topological surface area (TPSA), and xlog*P*.

More than 300,000 compounds were previously screened by qHTS for MBNL1-(CUG)_12_ inhibitors and represents the largest chemical library screened for DM1 to date [13]. Among these, the activity of five of these compounds has been characterized in previous studies and showed diverse effects in vitro [14,26]. In an effort to understand the properties (either structural or physicochemical) that confer inhibitory potency in these molecules, we performed a chemotype analysis by applying a PCA to the radial fingerprints. Fingerprints are usually chosen on a case-by-case basis through target validation, but in our study, radial fingerprints provided the most comprehensive results based on their performance. The projection of the radial fingerprints into the PCA space (σ^2^ = 41%; see Fig 1A) clearly demonstrates that active molecules share some properties and contain some privileged functional groups. Overall, the widespread distribution of data points was consistent with the wide structural diversity of the molecular set. Inactive molecules (red circles) and inconclusive data from the primary screening (orange) are scattered around the PCA space, while active molecules (blue) are focused on the origin. Not surprisingly, the superposition of previously reported active molecules which were not in the qHTS set, such as the triaminotriazine-acridine conjugates [17] and Hoechst 33258 [20], fall into the active region of the PCA subspace. Two molecules are slightly off, and these correspond to pentamidine (−1.1 and 0.8, for PC1 and PC2, respectively) and (*E*)-4-phenyl-2-(3-(thiophen-2-yl)acrylamido)thiophene-3-carboxylic acid (−3.7, 0.5 for PC1 and PC2, respectively); the latter was hypothesized to inhibit rCUG^exp^-MBNL1 interaction through protein inhibition, hence it was discarded as a RNA binder [26].

Fig 1B shows the distribution of the main physicochemical properties of the aforementioned active molecules. Overall, the molecular weights ranged between 30 u and 1120.8 u and charged molecules were not present. Hydrophobicity and polarity were evaluated with xlog*P* (−5.4 ≤ xlog*P* ≤ 8.2) and topological polar surface area (0 Å^2^ ≤ TPSA ≤ 467 Å^2^) respectively, and exhibited a normal distribution at around 2.9 Å^2^ and 103.7 Å^2^ respectively. Altogether, 94% of the chemical database conformed to the ‘Rule of five’ (RO5) guidelines. Interestingly, this procedure demonstrates that PCA is able to produce improved general models for heterogeneous data sets that can therefore be used for ligand-based selection of new potentially active compounds.

### Chemical library enumeration and description

We used two different cheminformatic approaches to select potentially bioactive compounds. On one hand, we selected compounds from our in-house chemical library according to their projection into the PCA space described above, selecting compounds similar to previously reported bioactive compounds. On the other hand, we chose compounds from a commercial database by using a combination of electrostatic potentials and shape complementarity to pentamidine.

**Fig 2:**
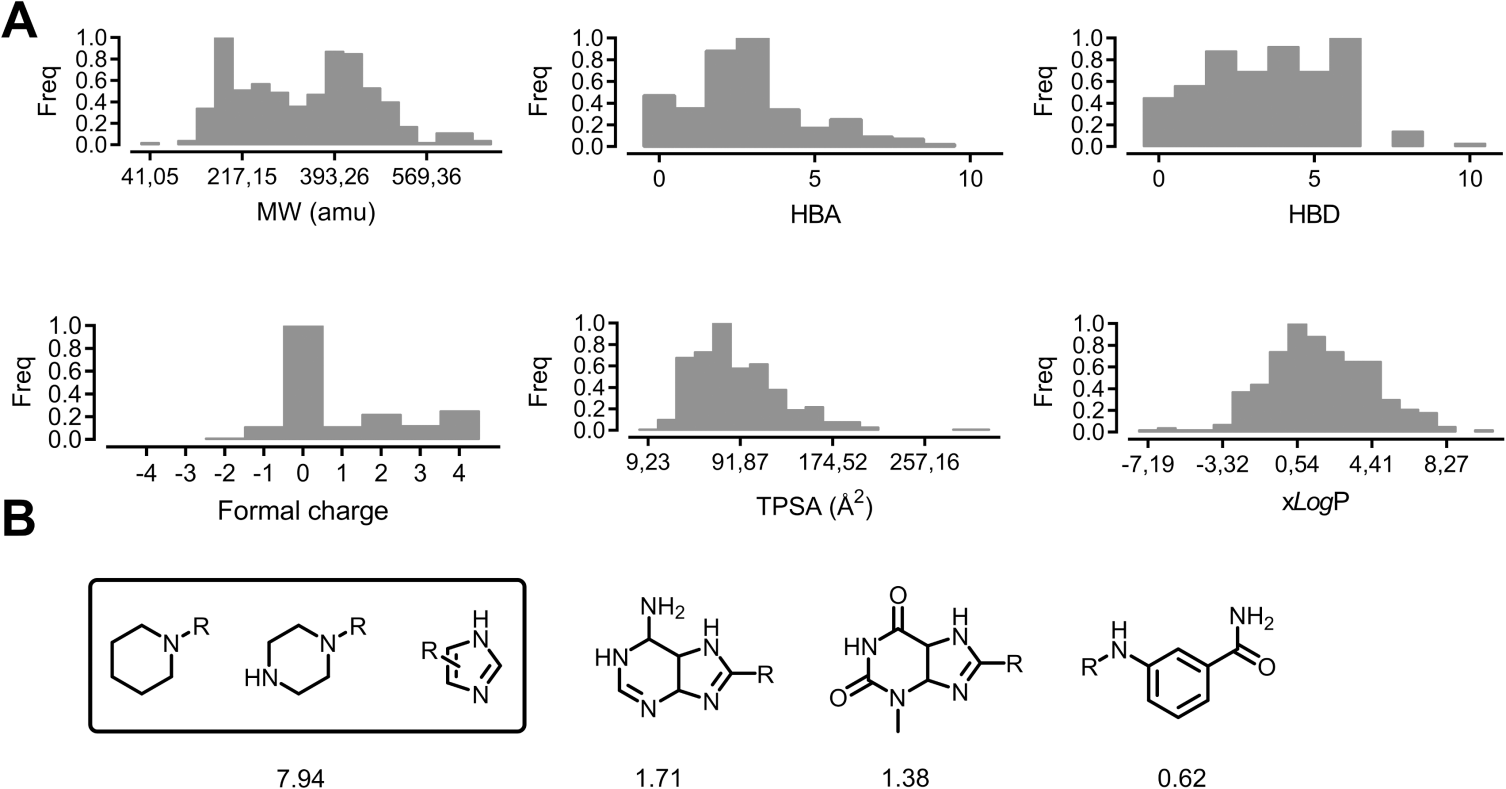
(A) Distribution of the principal physicochemical properties in our in-house chemical library containing more than 300 compounds: molecular weight (MW), hydrogen bond acceptors (HBAs), hydrogen bond donors (HBDs), formal charge, topological surface area (TPSA), and xlog*P*. (B) The most commonly observed fragments were ordered by their activity-adjusted frequency values (see Methods for a detailed description).

Chemicals were selected according to the chemotypes and molecular properties identified in the active compounds from a curated in-house chemical library containing more than 300 compounds. The physicochemical properties of the entire database are summarized in Fig 2A. The molecular weights ranged between 41.1 u and 710.2 u and hydrogen-bond acceptors and donors ranged between 0 and 10. The database contains some highly-charged molecules (in the [−4, 4] range), nevertheless, neutrally-charged ligands predominate, which should, theoretically, increase selectivity for the RNA at the expense of receptor affinity. Finally, xlog*P* (−7.2 ≤ xlog*P* ≤ 11.2) and topological polar surface area (9.2 Å^2^ ≤ TPSA ≤ 323 Å^2^) values exhibit a normal distribution around the mean values of 0.5 and 75.3 Å^2^ respectively. Altogether, 67% of our chemical database fulfils the RO5 guidelines.

Next, we analyzed the activity-adjusted frequency of the most common fragments among the screened database by using the retrosynthetic combinatorial analysis procedure [27] (RECAP, see Fig 2B). The highest frequency values are obtained for simple fragments such as piperidinyl, piperazinyl, and imidazolyl substituents (7.94%). In fact, the latter fragment is found in many of the reported DM1 bioactive structures, such as substituted benzimidazoles. In particular, bis-benzimidazoles have been extensively studied by Disney and co-workers and their bioactive potential have been clearly demonstrated [16, 28]. Other more complex fragments such as 4,5,6,7-tetrahydro-1*H*-purin-6-amine (1.71%), 3-methyl-3,4,5,7-tetrahydro-1*H*-purine-2,6-dione (1.38%), and *N*-substituted 3-methylbenzamide (0.62%) were also found, but yielded lower frequency values.

Our in-house chemical library contains a few scaffolds, mainly represented by substituted imidazoles and pyrido[2,3-*d*]pyrimidines, which are structurally similar to 3-phenylpyrimido[5,4-*e*][1,2,4]triazine-5,7(1*H*,6*H*)-dione, an active scaffold identified in the qHTS chemical library. Our compounds can be roughly divided into monomers and dimers, where two scaffolds are joined with a linker. While most of the monomers fall into the ‘active’ region described by the previous qHTS analysis (see Supporting Information, S2 Fig), the active regions of the dimers are displaced ‘south’, mainly because of their higher structural complexity (−1.5 < PC1 < 1.8; −25 < PC2 < 0). Both monomers and dimers close to the ‘active’ region and which passed the RO5 filters were selected for subsequent biological testing.

However, selecting ligands without including the receptor hampers the use of virtual screening strategies based on identifying the common features between active molecules or inferred from the receptor’s structure. Therefore, we decided to apply shape-based strategies that were proposed in a prospective study [16]) for hopping from toxic RNA-binders to new chemotypes. Based on the extensive bioactivity data available in the literature for pentamidine as a DM1 treatment, we enriched our chemical library with pentamidine-like structures. Using a previously described procedure [16], compounds were selected based on their chemical and structural similarities. This method has been successfully applied in previous DM1 studies which suggest that it is an excellent approach for improving potential hits or even for elucidating novel scaffolds. However, shape-based screening alone may not be sufficiently discriminatory because pentamidine only has a simple molecular framework. This fact, together with the prominent electrostatic features of the target RNA, motivated us to compare electrostatic potentials in addition to shape complementarity.

In this study we screened a lead-like commercial database containing more than 4 million compounds (250 ≤ MW ≤ 350, xlog*P* ≤ 3.5, rotatable bonds ≤ 7) (31); the 50 highest-scoring compounds were selected and analyzed accordingly. Similarity scores ranged between 1.16 and 1.35 (see Supporting Information, S1 Table). Next, we performed a diversity selection of the four most dissimilar molecules in terms of physicochemical properties (see Methods). These compounds were added to the in-house chemical library as our pentamidine-like subset.

**Fig 3:**
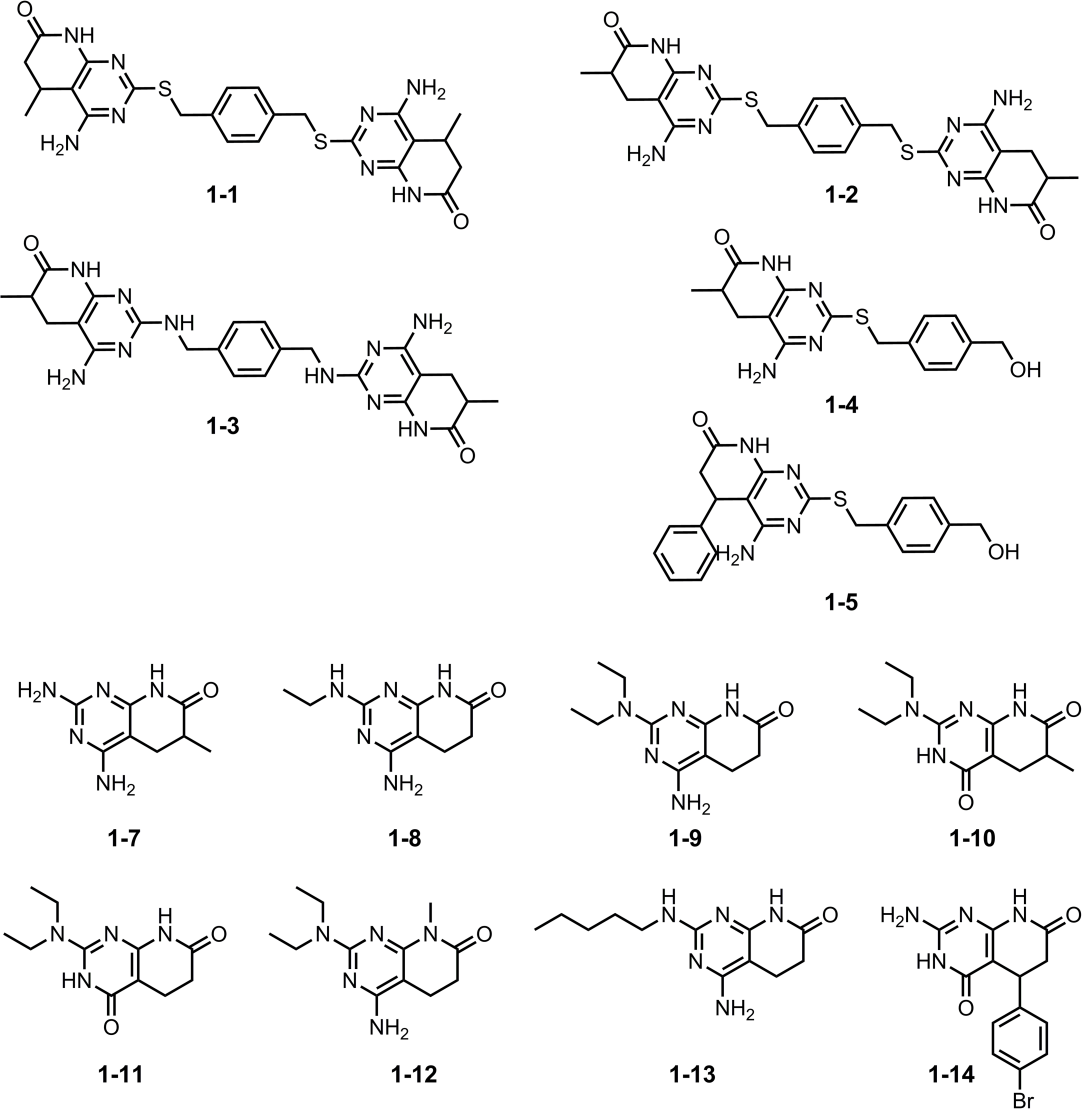
The molecular structure of the compounds we considered, based on the pyrido[2,3-*d*]pyrimidine scaffold (compound family 1). This family is divided into dimers (**1–1** to **1–3**) and monomers (**1–4** to **1–14**).

**Fig 4:**
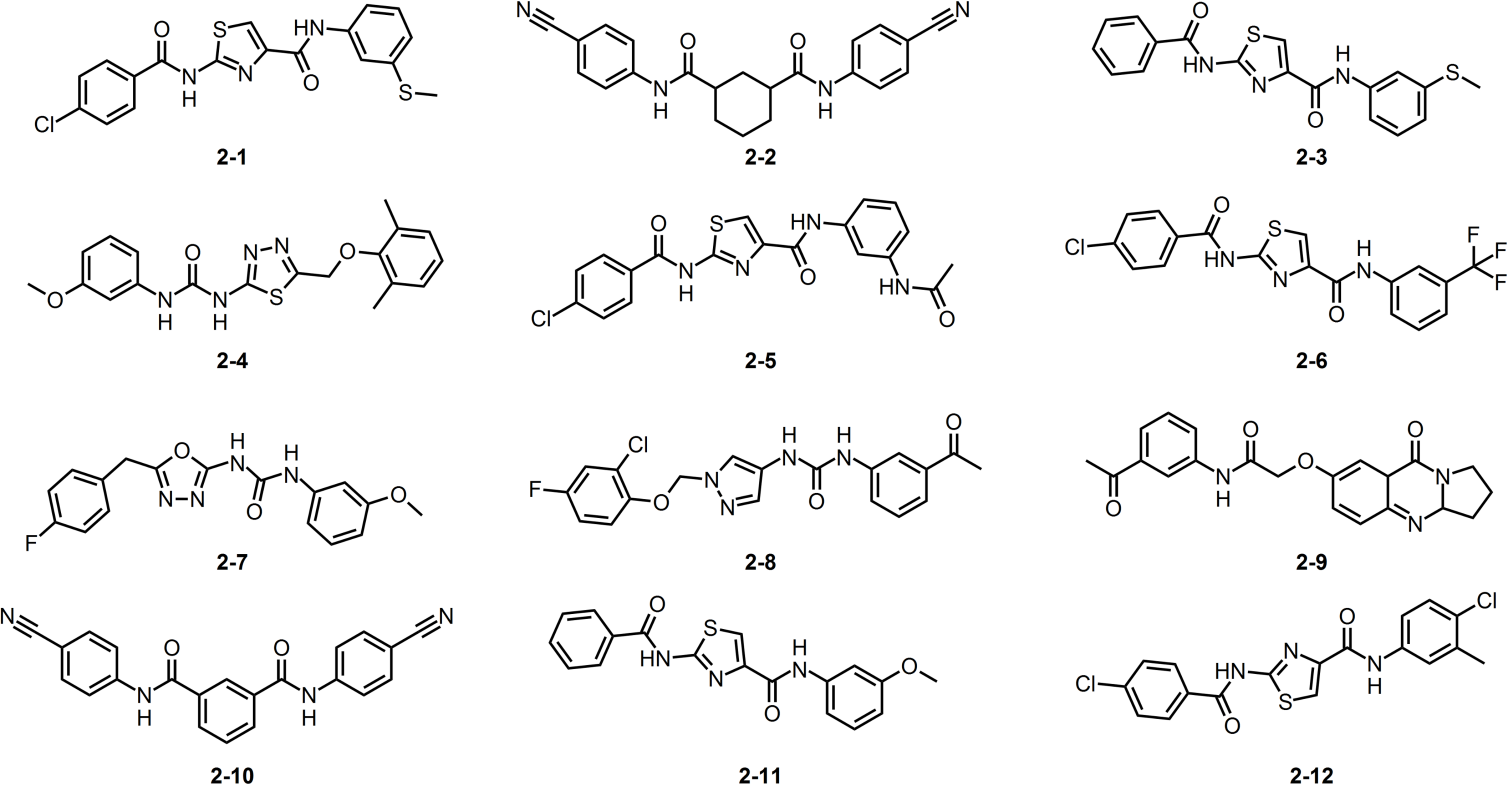
The molecular structure of pentamidine like compounds we considered (compound family 2).

In summary, a total of 23 compounds were selected, comprising 11 substituted pyrido[2,3-*d*]pyrimidines (compound family **1**, Fig 3) that locate inside the active region defined in the projected radial fingerprints space (blue region on Fig 1), and 12 pentamidine-like compounds (compound family **2**, see structures in Fig 4) selected by shape and electrostatic similarities. These compounds were then subjected to structure-based protocols, which require higher computational times.

### Rationalization of the CUG expanded-repeat druggability using molecular dynamics

In an effort to better understand of the chemotype requirements for RNA binding, we proceeded to investigate the druggability of the rCUG^exp^ receptor from a structural perspective. RNA-ligand recognition is a complex phenomenon involving many factors, but computational analyses of receptor druggable sites have been proven to correctly identify and rationalize structure-based drug design [29]. In this study, we rationalized rCUG^exp^ druggability using a MD-based approach previously described by Bakan et al., which enables binding sites to be identified using water and organic molecules as probes [29]. Each probe describes a different type of interaction, such as the presence of hydrophobic/hydrophilic binding sites or polar or charged areas, because of the differentiated physicochemical properties of the molecules used. Although the protocol was originally designed for proteins, we used it to study of the druggability of a truncated version of an rCUG^exp^ target based on the premise that RNAs may also contain binding surfaces similar to protein-binding pockets [30].

For this study, we investigated two independent systems: (**S1**) a system containing an r(CUG)_3_ structure in a TIP3P water box with Na^+^ counterions, and including a mixture of 70% isopropanol, 10% acetamide (polar group representative), and 20% sodium acetate-isopropylamine (charged group representative); (**S2**) an equivalent system with a composition of 30% isopropanol, 50% imidazole, 10% acetamide, 5% sodium acetate, and 5% isopropylamine. While system **S1** corresponds to a ‘standard’ composition for target druggability studies, the composition of system **S2** was optimized for investigating imidazole binding. Imidazole, and in particular benzimidazole, is a recognized chemotype that selectively binds to RNAs containing 1×1 internal loops produced by U-U non-canonical pairs [11,16,31].

**Fig 5:**
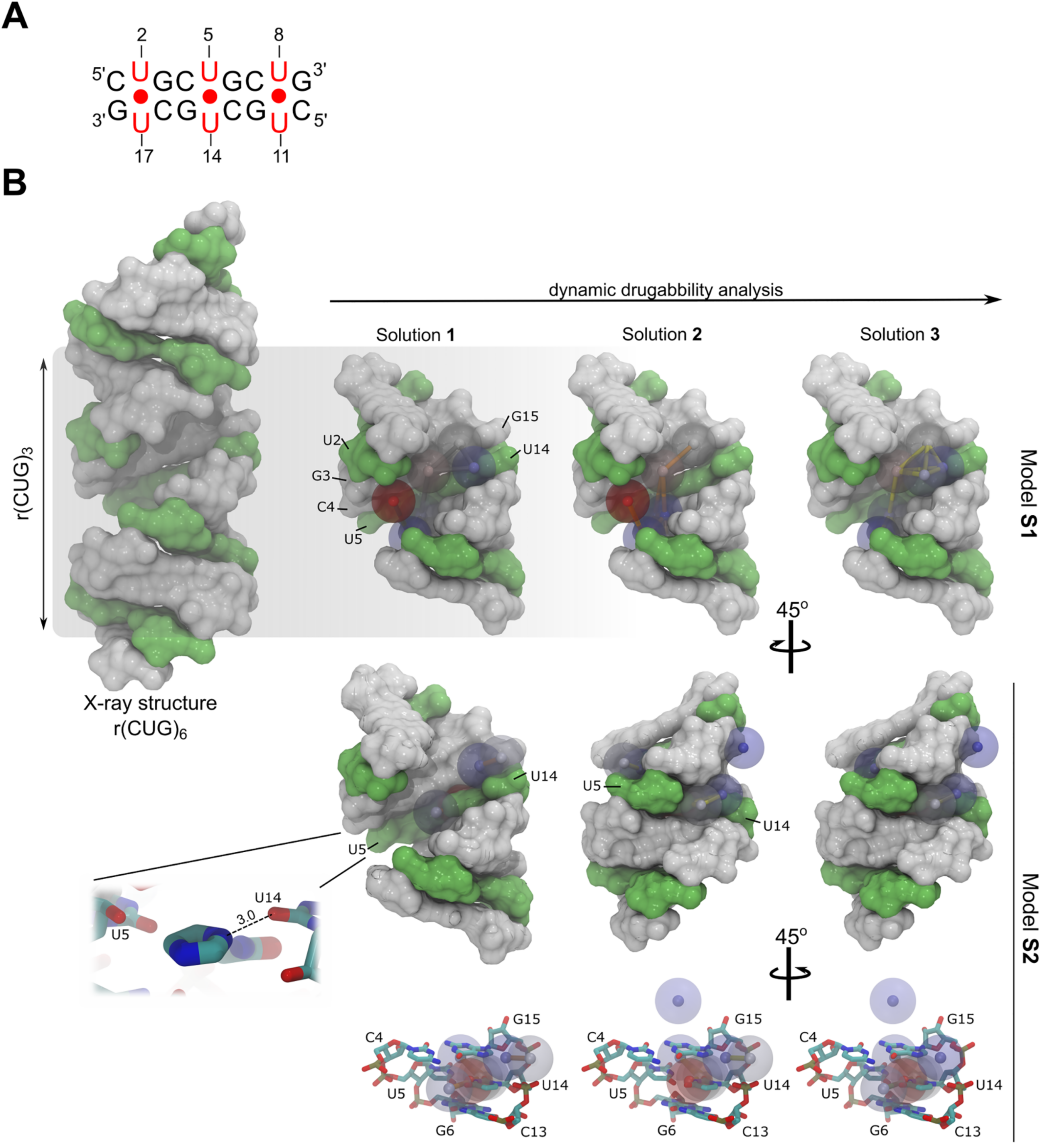
(A) Schematic representation of the r(CUG)_3_ model system. (B) Druggability analysis description of the **S1** and **S2** model systems. RNA is shown as a surface representation with C•G and G•C pairs in white and U-U pairs in green; three druggability solutions were obtained per system. Each druggable region, or hotspot, is represented by a colored sphere (red to blue, from lowest to highest binding-energy, respectively). Notice that the druggable sites in model **S1** are distributed along the major groove but are mainly located in the U-U pairs. Model **S2** had a stacking interaction pattern caused by the imidazole fragment, which stacks via one U-U pair and forms an H-bond with the O4 atom of U14 (3.0 Å). Some features can also be observed along the minor groove.

Druggability analysis performed using DruGUI software led to three druggable schemes for each system. The most probable druggable regions, or hotspots were analyzed (see Fig 5); red spheres correspond to the lowest energy hotspots (highest density probes) and blue spheres are the highest energy regions (lowest density probes). The highest proportion of small molecule binding consistently occurred through the major groove in system **S1**. However, the hotspots in **S1** are mainly located around the U-U non-canonical pairs (in particular U5 and U14 in our model system), which are the most dynamic and accessible regions in the RNA structure. This observation concurs with a recent MD study that describes a pocket along the groove produced by the intrinsic U-U pair dynamics [32]. Not surprisingly, hydrophobic and charged hotspots produced the highest affinities, yielding a binding-free energy of −1.66 kcal·mol^-1^ and −2.38 kcal·mol^-1^ respectively. The latter was defined by a charge-based interaction, as shown in solutions 1 and 2 of model **S1**, defined by interaction with the phosphate group on U8. The maximum achievable affinity for this site was 15 nM, which was mostly contributed by isopropanol probe interactions. Solutions 2 and 3 of system **S1** identified lower-affinity (23 nM and 80 nM, respectively) binding sites.

In sharp contrast with the first system, model **S2** was characterized by hotspots stacked into the middle of the U-U pair. The imidazole fragment accounts for more than 40% of the MD simulation stacked between U5-U14 and forms a unique hydrogen bond with the O4 atom on U14. Moreover, low energy regions are also found along the minor groove—these were evenly represented by hydrophobic and charged interactions and, at a lower level, polar interactions. The highest drug-like affinity in **S2** was lower than in model **S1** (37 nM for solution 1) but solutions 2 and 3 yielded regions with predicted affinities of 66 nM and 74 nM, respectively, which are in the same range as the **S1** model. Indeed, both druggability models provide feasible and high affinity solutions for rCUG^exp^ targeting (< 300 nM) and highlight that this particular RNA can recognize different types of interactions depending on the small molecule’s chemotype.

### Molecular recognition depends on essential dynamics

In silico studies of rCUG^exp^ have proven successful in previous de novo design strategies [33,34]. However, docking tends to yield poor complex predictions due to a lack of receptor flexibility, and MD requires high computational power in order to assess even relatively short virtual screening campaigns. Although MD is the most reliable method for macromolecule conformational sampling, sampling large-amplitude fluctuation events (such as conformational changes upon ligand binding) using this technique is still challenging. On the other hand, elastic network models (ENMs) and EDAs assume that the major collective modes of fluctuation dominate the functional dynamics [35]. In addition, the ability of this approach to correctly describe rCUG^exp^ dynamics near the equilibrium has recently been demonstrated [36]. Moreover, it has the advantage that the dynamics in different modes can be inspected and visualized individually. In this present study, we applied these assumptions in order to rationalize the potential bioactivity of the selected compounds from a structural perspective, in order to treat DM1 by coupling-docking and deformation of a r(CUG)_3_ target structure along its normal modes.

First, we constructed a dynamic ensemble by deforming the structure along the 20 lowest-frequency modes obtained from an all-atom model. Two deformations per mode were performed, up to a mass-weighted RMSD of 2 Å, hence a total of 40 RNA conformations were obtained (see Methods for details). By doing so, we explored the conformational space accessible to this RNA. Previous results suggested that a subset of 20 soft modes would be sufficient to map the most significant changes within the RNA structure [36]. In fact, this method enables ligands to ‘capture’ different RNA-small molecule complexes through conformational selection, thus a better description of the interaction can be achieved.

**Fig 6:**
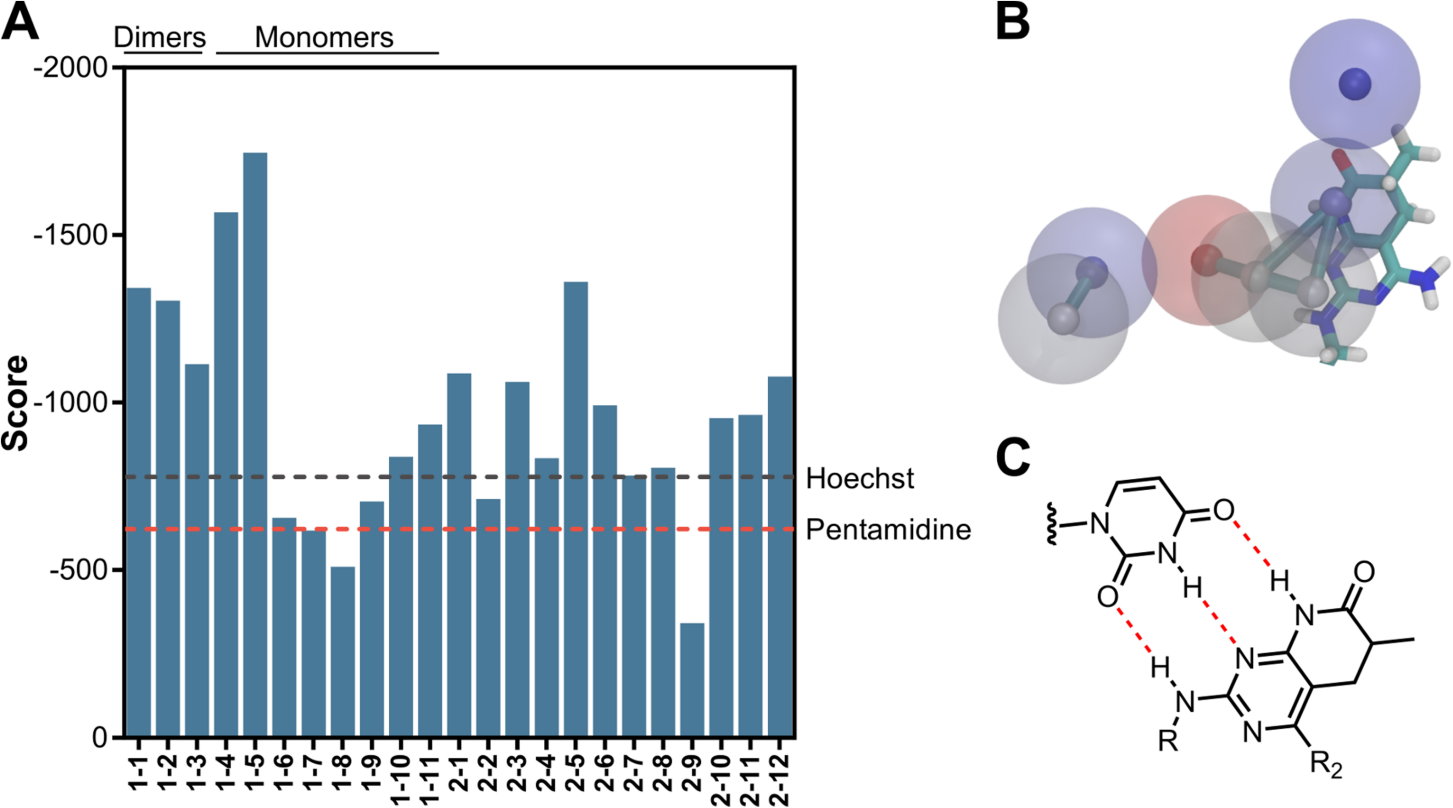
(A) LigandRNA score reported for compound families **1** and **2**. Pentamidine and Hoechst 33258 reference energies are included as red and grey lines, respectively. (B) Superposition of a pyrido[2,3-*d*]pyrimidine fragment and the binding hotspots (model **B**, solution 2) predicted with the druggability analysis. (C) Hypothesized interaction between a pyrido[2,3-*d*]pyrimidine subunit and a uracil residue.

Computationally inexpensive rigid-docking techniques perform poorly when benchmarking the predicted affinities against experimental data. However, simulation-based methods and comprehensive sampling of ligand and RNA conformational spaces may take entropic effects into account and improve binding-affinity predictions. Thus, we considered RNA flexibility into rigid docking by using the dynamic ensemble previously described. This led to 40 different RNA conformations representative of its dynamic behavior. Molecular docking was performed for every ligand, setting the number of output conformation to 50Thus, a total of 2000 potential bindings per compound were obtained. After re-scoring, the ligands were ranked according to the LigandRNA score obtained. Interestingly, the scores for our pyrido[2,3-*d*]pyrimidine dimers almost doubled those from our reference values (pentamidine and Hoechst 33258, see Fig 6A).

As in previous modelling studies [33], most of the conformations preferentially bind to the major groove of the rCUG^exp^, and more than three represented the lowest energy conformation in different RNA models (which enhances the chance of success in the conformational selection approach). In particular, the most energetically favorable conformations were from compound family **1** which bind through the RNA minor groove to form hydrogen bonds with U5-U14 and G6 (data not shown). The potential binding interactions occur in the non-canonical pairs via polar contacts and hydrogen bonding. Moreover, placement of the pyrido[2,3-*d*]pyrimidine subunit coincides with a predicted binding site highlighted by the druggability analysis for minor groove binding (model **S2**, solution 2, see Fig 6B). Hydrogen bond acceptors and donors from one pyrido[2,3-*d*]pyrimidine fall into the moderate- and low-affinity regions while the other interacts with the backbone through ribose interactions. Indeed, we hypothesize that family **1** compounds bind to RNA through a pattern of one triple hydrogen-bond per pyrido[2,3-*d*]pyrimidine subunit (Fig 6C).

Overall, these results correlate well with previous modelling studies (both molecular docking and MD) [26,30,37] and they help to shed some light onto the key interactions for small-molecule RNA recognition. A total of four compounds from family **1** (**1–1, 1–3, 1–4,** and **1–5)** and four compounds from family **2** (**2–3**, **2–5,** and **2–11**) were selected based on their predicted binding mechanism (higher docking scores) and their synthetic or commercial availability.

### Compounds 1–3 and 2–5 bind to CUG repeat RNA in vitro

We determined the in vitro affinity of the selected compounds to CUG repeats by performing fluorescence polarization spectroscopy experiments. A fluorescent (6-FAM-labeled) RNA probe containing 23 CUG repeats was incubated with increasing concentrations of the compounds to be tested. Whereas 6-FAM-CUG RNA molecules do not fluoresce in any particular polarization axis, binding to a molecule slows down the rotational movement of the molecule and increases its polarization values. In these experiments, we normalized the relative polarization to the values we obtained in the same experiment but using 100 μM pentamidine for comparative reasons. We noticed that, within the same concentration range, compound family 1 achieved higher fluorescence polarization than compound family 2 and showed clearer increases in polarization values as a dose-response to higher compound concentrations.

**Fig 7:**
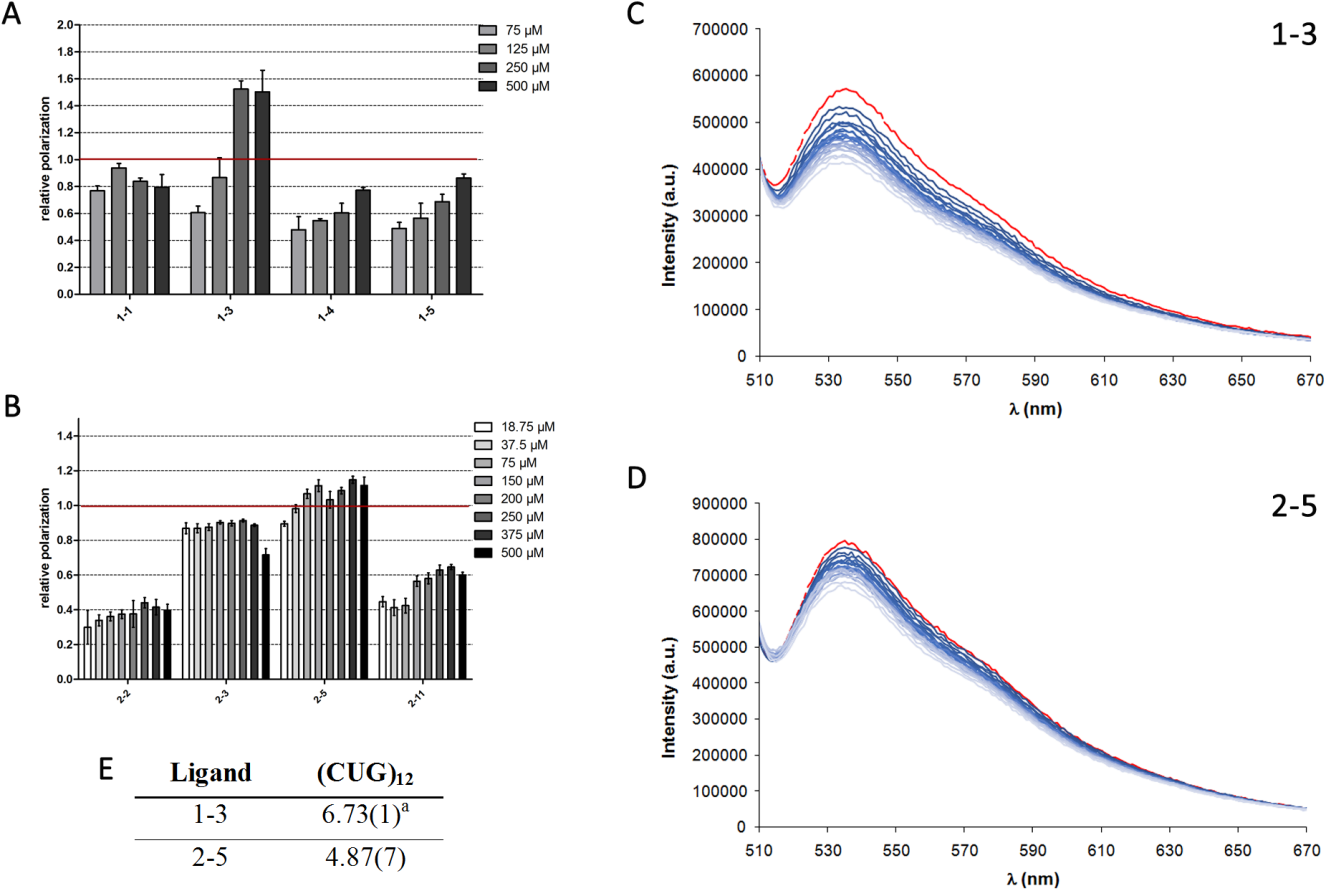
*In vitro* assays to evaluate the CUG-binding potential of compounds 1–3 and 2–5. Fluorescence polarization assays with the indicated concentrations of (A) four substituted pyrido[2,3-*d*]pyrimidines (compound family **1**) and (B) four pentamidine-like compounds (compound family **2**). Data were normalized to 100 μM pentamidine as a positive control (marked as a red line). Steady-state fluorescence emission profile for CUG^exp^-TO (red line) and changes in the emission intensity after addition of increasing amounts of (C) 1–3 and (D) 2–5 recorded in buffered NaCAC 50mM aqueous solution (pH = 7.4) at 298.1 ± 0.1 K. [TO] = 0.5 μM, [RNA] = 0.25 μM, [Ligand] = 0.1 mM.λ_exc_ = 485 nm. (E) Ligand-CUG^exp^ binding constants determined by displacement assays with TO. ^a^Values in parenthesis are standard deviations in the last significant figure.

One compound in each family achieved similar polarization values to pentamidine in the same range of concentrations: compounds **1–3** and **2–5**. Specifically, the fluorescence response could be measured for very low concentrations of compound **2–5**, possibly because the levels of binding-site saturation and bound CUG repeats were similar to those for pentamidine, but at far lower concentrations (e.g. 37.5 μM). On the other hand, the polarization values for compound **1–3** did not match those for pentamidine until they reached a concentration of 125 μM, and its binding to CUG repeats was not saturated until tested at a concentration of 250 μM (Fig 7A and 7B). Based on these experiments, we selected **1–3** and **2–5** for further evaluation because, compared to pentamidine, they showed the best polarization values and were representative of the two families of compounds. We estimated the binding constant for the interaction of selected ligands with CUG^exp^ by fluorescence spectroscopy using a thiazole orange (TO) displacement assay. Addition of any of the two ligands led to displacement of TO from CUG^exp^ with a consequent decrease in the fluorescence emission of TO (Fig 7C and 7D). The analysis of this titration permitted to estimate binding constants for the interaction of the ligands with CUG^exp^ (Fig 7E). Although both selected ligands interact with rCUG^ex,^, the values of the constants indicate a stronger binding of **1–3**.

### Compounds 1–3 and 2–5 release MBNL from foci in human myotonic dystrophy type 1 myoblasts

**Fig 8:**
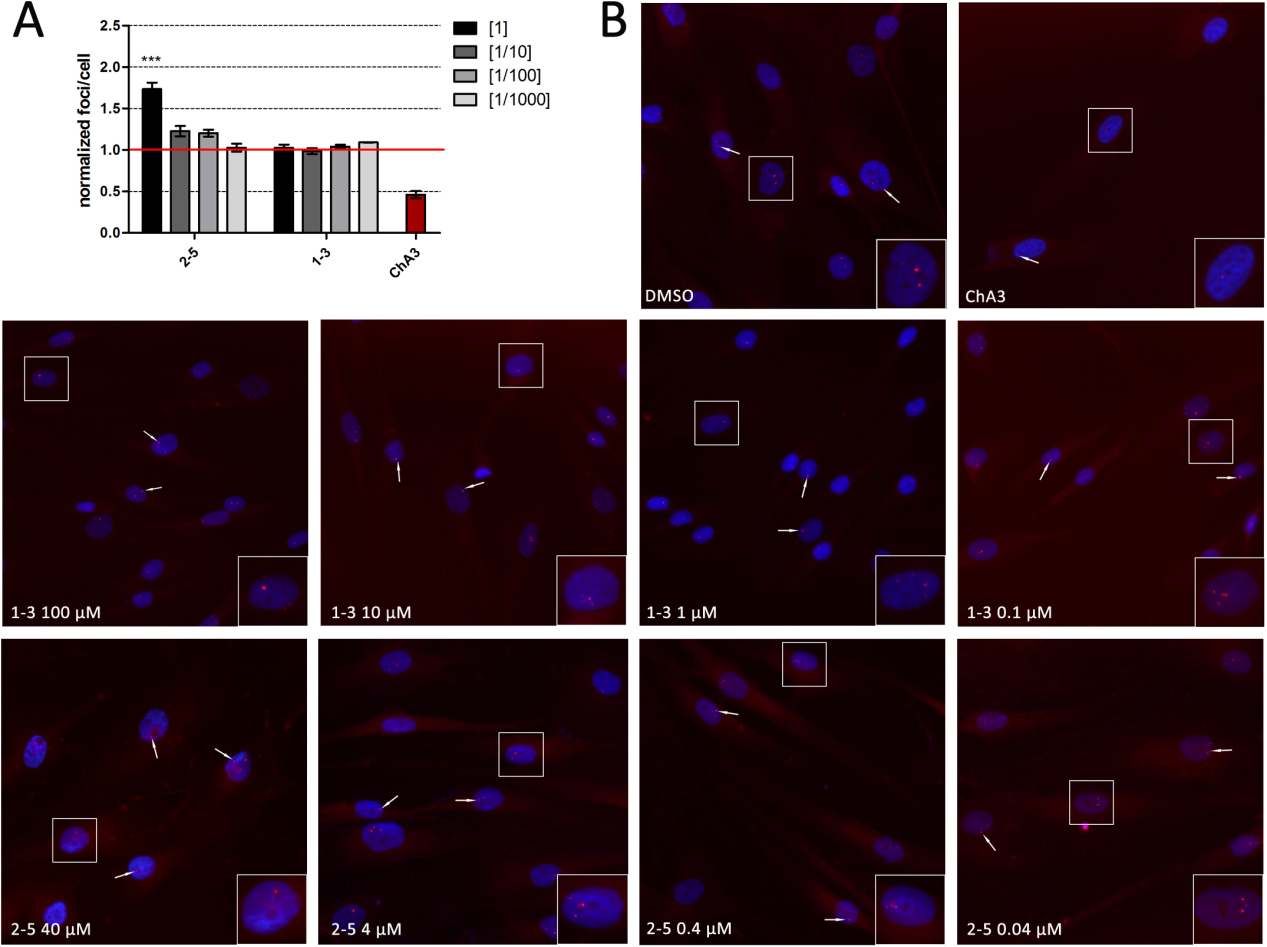
Quantification of the number of foci present after treatment with compounds 1–3 and 2–5. (A) Quantification of foci per cell from the analysis of a minimum of 1000 DM1 fibroblast nuclei in a range of four log dilutions starting at 40 μM for **2–5** and 100 μM for **1–3**. The results were normalized to DM1 cells treated with 1% DMSO (marked as a red line). (B) Representative fluorescence in situ hybridization (FISH) images showing foci in DM1 fibroblasts after 48 h treatment with a range of four log dilutions starting at 40 μM for **2–5** and 100 μM for **1–3**. 1% DMSO and 4 μM ChA3 were used as a negative and positive control, respectively. Nuclei were stained with Hoechst 33342 (blue) and rCUG^exp^ RNA foci were detected with a Cy3-labelled probe (red). Images included 2x enlargement of selected nucleus.

MBNL sequestration in CUG-RNA ribonuclear foci are histopathological hallmarks in DM1. To investigate whether compounds **1–3** and **2–5** had any impact on the number of foci present in a cell model of the disease, we performed fluorescence in situ hybridization (FISH) to detect CUG RNA accumulations in the nuclei of normal and DM1 fibroblasts. First, we studied the toxicity profile of these compounds in this cell type, obtaining an IC_10_ of 116 μM for compound **1–3** and 46.56 μM for **2–5**. Consequently, **1–3** and **2–5** were tested in fibroblasts at 100 μM and 40 μM, along with three serial log-dilution concentrations, respectively, for 48 h; 4 μM chromomycin A3 (ChA3), a drug that reduces foci number in DM1 fibroblasts [38] was used as a positive control (see Fig 8A and 8B). Whereas **1–3** had no effect on the number of foci at any of the concentrations, compound **2–5** tended to increase the number of foci, which was statistically significant at 40 μM.

To investigate whether these compounds had an effect on the subcellular localization of MBNL1 in human cells we immunodetected MBNL1 in normal and DM1 myoblasts grown with or without the **2–5** or **1–3** compounds. The amount of MBNL1 detected increased in the cytoplasm and in the cell nuclei of DM1 myoblasts grown either with 100 μM of **1–3** or 40 μM of **2–5,** compared to DM1 myoblasts exposed to 1% DMSO alone (solvent, Fig 8A). Taken together these results suggest that, although the interaction of these compounds with the CUG repeats is not sufficient to dissolve the foci, their interaction with the RNA does release MBNL into the nuclei and cytoplasm.

### Compounds 1–3 and 2–5 improve locomotion defects in myotonic dystrophy type 1 model flies

**Fig 9:**
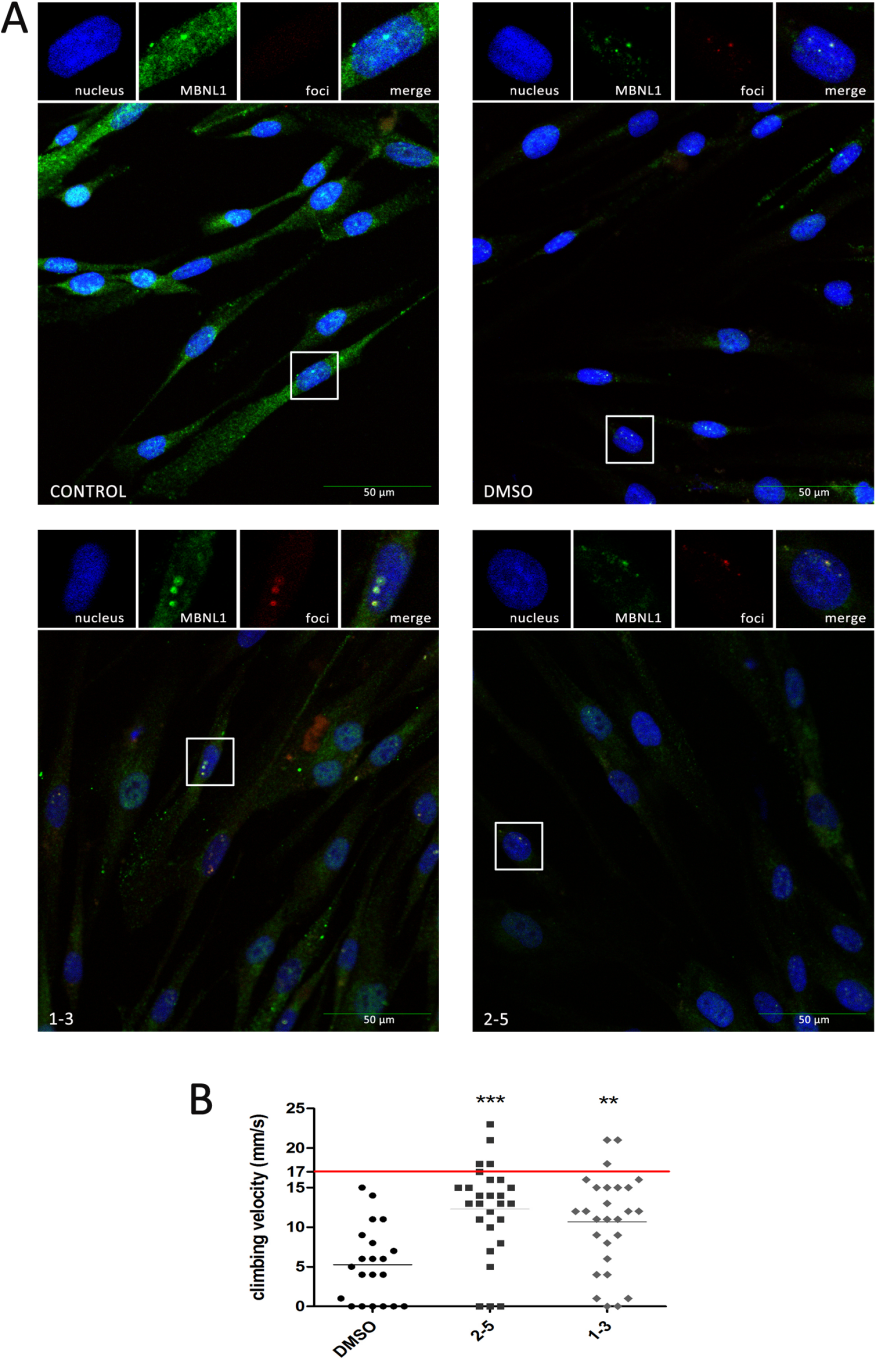
Subcellular distribution of MBNL1 and in vivo evaluation of therapeutic potential. (A) Confocal images of co-localization of MBNL1 (green) and foci (red) in DM1 myoblasts. Cells were treated with compounds for 48 h; nuclei were counter-stained with DAPI (blue). 1% DMSO-treated DM1 myoblasts and normal myoblasts were used as controls. Images include 2x enlargement of selected nucleus. (B) Climbing assay performed on 30 adult male flies fed for five days with the indicated compound. Climbing velocity was significantly higher in flies receiving 40 μM compound **2–5** or 100 μM compound **1–3** compared to DM1-model flies receiving only 1% DMSO solvent carrier. The average climbing velocity for wild type flies is marked as a red line. Statistics were calculated using the Student's t-test (**p < 0.01, ***p < 0.001).

To test whether the MBNL protein released by the compounds was sufficient to exert a therapeutic effect in vivo, we used transgenic flies that express non-coding 480 CTG repeat RNA throughout their musculature and thus, reproduce DM1-like phenotypes, chiefly muscle atrophy and locomotion defects (Bargiela et al. [39] and unpublished observations). Using a living organism is the first step in drug evaluation because it provides a system for simultaneously testing the absorption, distribution, metabolism, excretion, and toxicity (ADME-Tox) parameters of a compound. Flies were fed compound **1–3** or **2–5** at a concentration of 100 μM or 40 μM, respectively, for five days after hatching. Whereas climbing speed of flies expressing CTG repeats throughout their muscles was strongly impaired (around 5 mm/s) compared to the wild-type speed (17 ± 0.7 mm/s), oral administration of compounds **1–3** or **2–5** robustly rescued the impaired climbing speed for DM1 flies, more than doubling them (Fig 9B). Thus, these data support the therapeutic potential of both compounds.

## Discussion

Finding small molecules that selectively bind and recognize RNA target structures is a challenging process, hence the compilation of RNA-focused libraries has recently been proposed [28]. Specifically, DM1 is one of the most well-studied diseases produced by an RNA gain-of-function pathomechanism and several chemotypes that avert some of its phenotype features have been reported [11,20,28,38,40,41].

In this current study we applied several drug design techniques to identify chemotypes with potential activity against DM1; ligand and structure-based drug design strategies were combined which allowed the development and identification of new drug candidates for the treatment of this disease. It is also worth noting that we applied ligand-based approaches to establish a strategy for enriching the compound selection. Importantly, a precedent for evaluating structural similarities between active and non-active compounds using radial fingerprints in a qHTS-derived database has been reported in the literature [13]. Interestingly, our PCA identified a strong correlation between compound activity and the PCA projection; although the first two principal components only accounted for 41% of the explained variance, projection into this two-dimensional space was sufficient to enclose the active molecules in a well-defined region. Unfortunately, the active-labeled region is not exclusive and PCA projection cannot lead to unequivocal classification. Thus, this model can be easily used to increase the enrichment-factor in virtual screening of potential drug candidates, although with the caveat that the power of PCA analysis not be overstretched in this context.

We also used shape and electrostatic complementarity screening to demonstrate an effective procedure for improving the chances of targeting the relevant RNA, allowing potentially active pentamidine-like compounds to be identified as repurposed drugs. Among these, we selected two families of compounds: compound family **1**, consisting of substituted pyrido[2,3-*d*]pyrimidines, and compound family **2** comprising a heterogeneous group of pentamidine-like commercial compounds. Because this strategy identified many candidates, we applied structure-based drug design techniques in order to obtain a list of prioritized compounds for purchase or synthesis. When synthesized, compound purity was confirmed by NMR before testing.

The lack of a specific active site in RNA structures hampers selective recognition of small molecules, therefore, the druggability analysis was decisive in identifying the preferred accessible regions of the rCUG molecule. On the one hand, highly charged and hydrophobic compounds should potentially bind through the major groove of the RNA through specific U-U interactions; on the other hand, Hoechst-like or imidazole-containing compounds usually stack around the U-U pairs or bind to the RNA minor groove, as in the previously elucidated DNA-Hoechst X-ray crystal structure [42]. Thus, we applied this MD-based method to assess druggability; our results potentially explain the selectivity of specific chemotypes, such as imidazole derivatives, which presumably act via a combination of stacking interactions and specific hydrogen bonding patterns with the U-U pairs.

By coupling MD simulations with a structural study on the druggability of rCUG^exp^ systems, we identified two chemotypes that bind to the RNA. Furthermore, in vitro fluorescence polarization spectroscopy confirmed that some compounds belonging to these chemotypes have a pentamidine-like affinity for CUG repeats. We then tested one representative compound each from group **1** and **2** in DM1 model cells in order to test their effects of on the histological hallmarks of the disease; compound **1–3** did not modify the number of ribonuclear foci in DM1 fibroblasts, whereas compound **2–5** increased these RNA foci accumulations.

Although CUG-binding compounds typically reduce the presence of foci, some observations suggest that an increase in muscleblind expression may lead to an increase in foci. In particular, Houseley et al. reported that overexpression of muscleblind protein isoform A stabilizes cytoplasmic CUG-repeat RNA, which could further contribute to cellular ribonuclear foci accumulation [43]. Similarly, Yu et al. found that co-expression of human MBNL1 isoforms and CUG-repeat RNA in Drosophila photoreceptors leads to cleavage and concurrent upregulation of the levels of these RNA-repeat transcripts [44]. Importantly, Hoskins et al. reported that although dilomofungin (a MBNL1-CUG RNA binding inhibitor) treatment reduced CUG-expansion turnover and produced a large increase in the number of ribonuclear foci, it also led to a significant recovery in *SERCA* transcript missplicing [45].

Similarly, we observed that compound **2–5** could bind CUG repeats at a concentration of 40 μM but that this binding resulted in an increased number of foci rather than the expected reduction. We hypothesize that the affinity of compound **2–5** to the CUG repeats at this concentration might have caused conformational changes in the CUG hairpin, allowing the release of MBNL observed. We also detected free MBNL as a response to compound **1–3**, although in this case polarization assays showed that the concentration required was proportionately higher than that required by for **2–5**, and so, lower concentrations of **1–3** are not expected to have an effect on MBNL release.

In both cases, the amount of MBNL released was sufficient to at least partially rescue the functional phenotype of DM1 model flies, as shown by strong improvements in their climbing velocity (double compared to control flies) when fed either **1–3** or **2–5**. In addition, because we, and others, have shown that the number of foci present in DM1 model cells increases in the presence of these compounds despite MBNL release, it is likely that a reduction in CUG RNA is not required for the functional improvement in DM1 phenotypes seen in these flies. Thus, in this study we have confirmed the biological activity of new chemotypes identified in our in silico studies, which represent a valid and useful strategy for high-throughput screening of anti-DM1 compounds.

## Methods

### Chemoinformatics analysis of the chemical space

Compound collections were analyzed and compared based on physicochemical properties, scaffolds, and radial fingerprints. Physicochemical properties were computed using the FILTER application from the OpenEye suite (version 2.1.1, http://www.eyesopen.com) [46]. To obtain a visual representation of the molecular space, a principal component analysis (PCA) was carried out using Canvas from the Schrödinger 2014 suite [47,48], considering the radial fingerprints properties. Scaffold analysis was conducted using Scaffold Hunter (Supporting Information, S1 Fig) [49].

Similarity screening was performed with the ROCS tool from the OpenEye suite [50]. The chemical library was selected from a subset of the ZINC database (clean lead-like, 250 ≤ MW ≤ 350, xlog*P* ≤ 3.5 and rotatable bonds ≤ 7) [51]. First, 100 conformers per molecule were generated using Omega (version 2.02) [52], including the query structure pentamidine. ROCS was used for three-dimensional shape comparison, and the 100 molecules with the highest shape Tanimoto index values were output in rank order as hits. For electrostatic comparison (EON 1.1) [53], a single lowest-energy conformer of pentamidine was used for all comparisons to re-rank the top 50 shape-based hits. A complete list of the selected compounds is available in the Supporting Information (S1 Table).

To finally reduce the number of compounds to synthesize, diversity selection was performed on a chemical space described by 2D structural and physicochemical molecular descriptors. A set of 128 attributes were computed with MOE 2014.09 software [54]. Genetic algorithms were applied for maximizing the minimum intermolecular distance to obtain the most dissimilar set of 50 compounds. Diversity selection was performed using PRALINS software [55].

The 12 most dissimilar compounds were selected, using the Euclidean distance metric.

### Molecular dynamics simulations for druggability assessment

Available (CUG)_n_ structures were retrieved from the Protein Data Bank (PDB; ids: 1zev [56], 3gm7 [57], 3syw [58], 3szx [58], 4e48 [59], 4fnj [60]). Previous computational studies suggested that a three-nucleotide repeat expansion should suffice for in silico studies of small molecules [35]. Structures were prepared using PyMOL [61] by retaining only fragments with three repeats (n = 3). For longer repeated fragments, all the possible n = 3 combinations were extracted as individual structures. Next, all the structures were superimposed and saved as a *pdb* ensemble. Simulations were performed using NAMD [62] software and the CHARMM force field [63]. Productive simulation times were 40 ns in all runs. The druggability was assessed using a molecular dynamics (MD) approach which was performed using DruGUI software [29]. Two different sets of probes were used: (**A**) a system with a mixture of 70% isopropanol, 10% acetamide, and 20% acetate—isopropylamine; (**B**) an equivalent system with a composition of 30% isopropanol, 50% imidazole, 10% acetamide, 5% acetate, and 5% isopropylamine.

### Structural dynamics of the CUG expanded-repeat fragments

EDA was completed with ProDy [64] using a total of 20 deformation modes and using the previous r(CUG)_3_ ensemble. A total of 40 RNA conformations were obtained by applying two deformations per mode using a mass-weighted root-mean-square deviation (RMSD) of 2 Å.

### Molecular docking

Molecular docking was performed according to the cross-docking approach. The compound structure was energy minimized using MOE (molecular operating environment) software by defining the MMFF94 force field [65] and calculating the AM1 charges [66]. Molecular docking was conducted using Vina software (version 1.1.2) [67]. A total of 20 conformations were generated per run which were re-scored using the LigandRNA [68] server and ranked according to the score value.

### Fluorescence polarization assay

Carboxyfluorescein (6-FAM)-labeled CUG RNA (23 CUG repeats; 6-FAM-CUG_23_) at 6 nM was incubated with compounds at different concentrations in binding buffer (50 mM Tris-HCl pH 7.0, 250 mM NaCl, 50 μM ZnCl_2_, 10% glycerol, and 1 mM DTT) on ice for 20 min in the dark. Fluorescence polarization was measured in an EnVision^®^ Multilabel Reader using a FP480 excitation filter and an FP535 emission filter. The readings for each concentration were quadruplicated for all the compounds.

### Displacement assay

The emission spectra were recorded with a PTI MO-5020 spectrofluorimeter in the 510–670 nm range with excitation wavelength of 485 nm at 298.1 ± 0.1 K. (CUG)_12_ RNA stock solutions RNase-free water and thiazole orange (TO) stock solutions were prepared in DMSO. Ligand stock solutions (**1–3** and **2–5**) were prepared in DMSO (100 mM) and then diluted solution of the ligands (0.1 mM) were prepared in buffered sodium cacodylate (NaCAC) 50 mM (pH = 7.4) aqueous solution.

Typically, 2.5 μL (100 μM) of RNA (2.5 x 10^−10^ mols) was incubated with 1μL (500 μM) of TO (5 x 10^−10^ mols) in 1mL of NaCAC 50mM buffered solution, pH = 7.4 for 5 minutes. The fluorescence of the solution was recorded and then increasing amounts of ligand (0.1 mM) were added until a final ratio TO:ligand 1:10 was reached. The solutions were stirred and their fluorescence was measured after 2 min of equilibration time. Dissociation constants are reported as the average of at least two independent measurements. Previously to the displacement assays, the binding constants of TO with CUG was determined by direct fluorescence titration being 6.5(1) logarithmic units. The values of the constants were calculated with the program HypSpec [69].

### Cell culture conditions

The cell model of the disease (provided by D. Furling’s laboratory in the *Institute of Myologie*, Paris) consisted of healthy and DM1 (1300 CTG repeats) immortalized (hTERT) skin fibroblasts conditionally expressing MyoD. Fibroblast cells were grown in Dulbecco’s Modified Eagle Medium (DMEM) with 4.5 g/L glucose, 1% penicillin and streptomycin (P/S), and 10% fetal bovine serum (FBS; Sigma). To transdifferentiate fibroblasts into myoblasts by inducing MyoD expression, the cells were plated in muscle differentiation medium (MDM) containing DMEM with 4.5 g/L glucose, 1% P/S, 2% horse serum, 1% apo-transferrin (10 mg/ml), 0.1% insulin (10 mg/ml), and 0.02% doxycycline (10 mg/ml).

### Double MBNL staining and FISH

Given the low levels of MBNL expression in fibroblasts, double MBNL staining and FISH to detect ribonuclear foci was performed in fibroblasts transdifferentiated to myoblasts (6 days in MDM).Fibroblasts were aliquoted into 24-well plates (4×10^4^ cells per well) and changed to MDM media the following day. After 4 days in MDM, the compounds were added and the plates were incubated for further 48 h. Subsequently, the cells were fixed in 4% paraformaldehyde (PFA) for 10 min at room temperature followed by several washes in 1× PBS. Cells were then permeabilized with 0.3% Triton in PBS (PBT), blocked (PBT with 1% donkey serum and 0.5% BSA) for 30 min at room temperature, and incubated with primary antibody (mouse anti-MBNL1 1:200; Sigma) at 4°C overnight. After several washes with PBT the cells were incubated for 45 min with biotin-conjugated secondary antibody (Sigma) at a 1:200 dilution. Cells were then incubated with ABC solution (ABC kit, VECTASTAIN) for 30 min at room temperature, followed by PBT washes and incubation with streptavidin-FITC (1:200) for 45 min. After several washes in 1 x PBS, cells were incubated in hybridization buffer during 30 min at room temperature and subsequently incubated during 2 hours with the Cy3-CAG10 probe(1:500) in hybridization buffer at 37°C. Finally, probe excess was removed by two consecutive washes in 2 x SSC (at 37°C) and 0,5 x SSC (at room temperature). Cells were mounted with VECTASHIELD^®^ mounting medium containing DAPI (Vector) to detect the nuclei. Images were taken using an Olympus FluoView™ FV1000 confocal microscope.

### Foci detection

Fibroblasts were aliquoted into 96-well plates (1.0×10^4^ cells per well), incubated with the compounds (48 h) and fixed in 4% PFA for 10 min at room temperature followed by several washes in 1× PBS. Fixed cells were incubated in pre-hybridization buffer (2× SSC, 30% deionized formamide) for 10 min at room temperature and hybridized with Cy3-(CAG)_7_ -Cy3 labelled probe diluted 1:500 in hybridization buffer (40% formamide, 2× SSC, 0.2% BSA, 10% dextran sulfate, 2 mM ribonucleoside-vanadyl complex, 10% tRNA [10 mg/ml], and 10% herring sperm) for 2 h at 37°C. After hybridization, we washed the cells twice with pre-hybridization buffer for 15 min at 45°C, washed twice with 0.5× SSC for 5 min at 37°C, washed with 1× PBS for 15 min at room temperature, incubated them with Hoechst 33342 (5 mg/ml) diluted 1:2000 in 1× PBS for 20 min at room temperature, and mounted them with 20% Mowiol. Images were taken and analyzed using an IN Cell Analyzer 2200 Imaging System.

### Climbing assay

To assess any improvements in the locomotion defects typical of DM1 flies, we transferred newly emerged flies expressing 480 CTG repeats under the myosin heavy chain (Mhc)-Gal4 driver expression pattern, which shows general expression in Drosophila musculature [9], to tubes containing the compound under study or 1% DMSO dissolved in standard nutritive media. The flies were kept in these experimental conditions for an additional 5 days under standard storage conditions. Two groups of 15 five-day-old males per experimental condition were transferred into 1.5 cm diameter, 25 cm long disposable pipettes to assess their climbing speed. The height reached by each fly during a period of 10 s was recorded with a camera.

## Supporting information

S1 Fig Scaffold tree map (molecular frameworks) derived from Scaffold Hunter. Only the highest complexity scaffold is shown for each cluster along with the SMILES code for each substructure. The space of each scaffold is filled according to their activity score.
(TIFF)

S2 Fig Projection over the PC1 and PC2 subspace from the radial fingerprints of complete qHTS subset (left) and in-house pyrido[2,3-*d*]pyrimidines (right). The coloured regions correspond to the ‘active’ region.
(TIFF)

S1 Table Top 50 molecules obtained from the ZINC database using the EON protocol as described in the Materials and Methods section. (PDF)
