# Supplementary figures and images for: In silico discovery of substituted pyrido[2,3-d]pyrimidines and pentamidine-like compounds with biological activity in myotonic dystrophy models
Source: PLoS One. 2017 Jun 5;12(6):e0178931. doi: 10.1371/journal.pone.0178931 (PMC5459475; doi:10.1371/journal.pone.0178931)

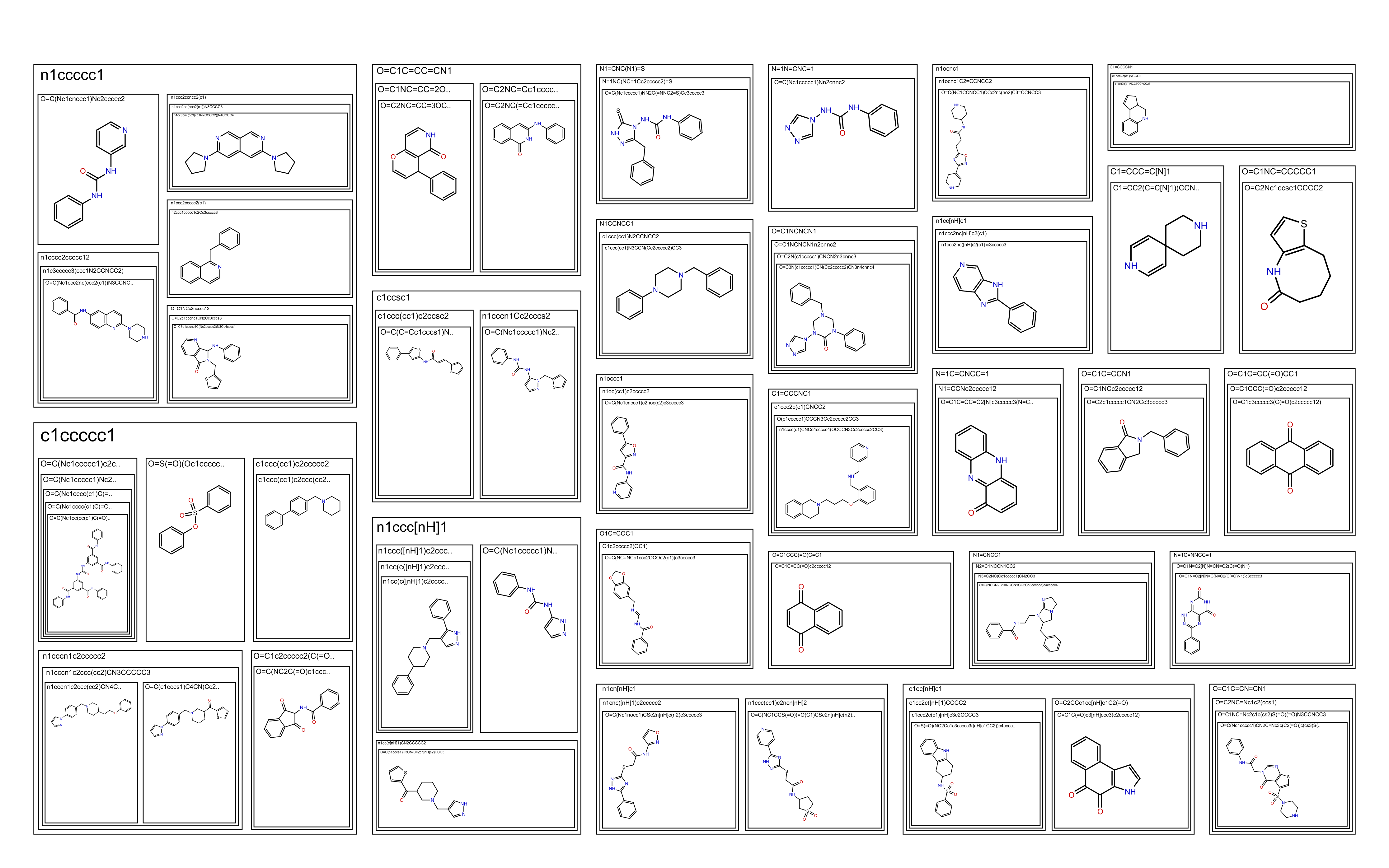

Supplement: S1 Fig — Only the highest complexity scaffold is shown for each cluster along with the SMILES code for each substructure. The space of each scaffold is filled according to their activity score. (TIFF) [file pone.0178931.s001.tiff]
