# Supplementary material for: In silico discovery of substituted pyrido[2,3-d]pyrimidines and pentamidine-like compounds with biological activity in myotonic dystrophy models
Source: PLoS One. 2017 Jun 5;12(6):e0178931. doi: 10.1371/journal.pone.0178931 (PMC5459475; doi:10.1371/journal.pone.0178931)
Supplement: S1 Table — (PDF) [file pone.0178931.s003.pdf]

In silico discovery of substituted pyrido[2,3-*d*]pyrimidines and pentamidine-like compounds with biological activity in myotonic dystrophy models

Àlex L. González[1]‡, Piotr Konieczny[2,3,4]‡, Beatriz Llamusi [2,3,4], Estefanía Delgado-Pinar[5], José I. Borrell[1], Jordi Teixidó[1], Enrique García-España[5], Manuel Pérez-Alonso[2,3,4], Roger Estrada-Tejedor[1]*, Rubén Artero[2,3,4]

‡ Equal contributors

[1]Grup d'Enginyeria Molecular (GEM), Institut Químic de Sarrià (IQS) – Universitat Ramon Llull (URL), Barcelona, Catalonia, Spain

[2]Translational Genomics Group, Incliva Health Research Institute, Valencia, Spain

[3]Department of Genetics and Interdisciplinary Research Structure for Biotechnology and Biomedicine (ERI BIOTECMED), University of Valencia, Valencia, Spain

[4]Incliva-CIPF joint unit, Valencia, Spain.

[5]Instituto de Ciencia Molecular, Universidad de Valencia, Paterna, Valencia, Spain.

S1 Table. Top 50 molecules obtained from the ZINC database using the EON^2 protocol as described in the Materials and Methods section.

ZINC ID	ET_pb	ET_coul	ET_combo	EON_ShapeTanimoto	Rank
ZINC08873523	0.775	0.74	1.569	0.794	1
ZINC60135435	0.701	0.643	1.562	0.861	2
ZINC08873186	0.754	0.705	1.535	0.781	3
ZINC01248815	0.672	0.627	1.524	0.852	4
ZINC08873550	0.738	0.681	1.52	0.782	5
ZINC08873397	0.705	0.676	1.511	0.806	6
ZINC38551188	0.68	0.674	1.506	0.826	7
ZINC32610238	0.661	0.7	1.505	0.845	8
ZINC12902935	0.662	0.698	1.505	0.843	9
ZINC06926130	0.632	0.623	1.503	0.872	10
ZINC08873084	0.723	0.668	1.502	0.78	11
ZINC08873462	0.72	0.686	1.496	0.776	12
ZINC12902559	0.666	0.701	1.494	0.828	13
ZINC08873463	0.719	0.681	1.492	0.773	14
ZINC38551234	0.684	0.641	1.49	0.806	15
ZINC08914133	0.731	0.711	1.489	0.758	16
ZINC09318666	0.692	0.645	1.488	0.796	17
ZINC08873391	0.707	0.672	1.488	0.781	18
ZINC60135037	0.642	0.569	1.487	0.845	19
ZINC08873383	0.707	0.674	1.487	0.78	20
ZINC08873405	0.689	0.652	1.486	0.797	21
ZINC06876442	0.681	0.677	1.484	0.803	22
ZINC08873379	0.706	0.671	1.482	0.776	23
ZINC39475841	0.644	0.62	1.482	0.837	24
ZINC08873077	0.692	0.633	1.48	0.788	25
ZINC12898913	0.684	0.719	1.479	0.795	26
ZINC08394670	0.681	0.669	1.477	0.796	27
ZINC08873460	0.703	0.662	1.476	0.773	28
ZINC73348308	0.717	0.638	1.475	0.758	29
ZINC08873122	0.74	0.701	1.473	0.732	30
ZINC08873215	0.731	0.704	1.47	0.739	31
ZINC08873444	0.69	0.642	1.465	0.775	32
ZINC38551244	0.652	0.623	1.463	0.811	33
ZINC08739102	0.675	0.637	1.462	0.787	34
ZINC12671768	0.619	0.633	1.46	0.841	35
ZINC08873112	0.705	0.676	1.459	0.754	36
ZINC08873363	0.68	0.633	1.459	0.778	37
ZINC60135528	0.678	0.597	1.457	0.78	38
ZINC08873109	0.703	0.675	1.457	0.754	39
ZINC60135285	0.636	0.578	1.456	0.82	40
ZINC70845249	0.668	0.592	1.456	0.788	41
ZINC08873110	0.704	0.674	1.454	0.75	42

Continuation of table S1.					
ZINC08873439	0.685	0.629	1.454	0.769	43
ZINC04575969	0.591	0.57	1.452	0.861	44
ZINC08873136	0.694	0.642	1.451	0.757	45
ZINC08873449	0.68	0.632	1.451	0.77	46
ZINC58004049	0.727	0.683	1.449	0.722	47
ZINC03065518	0.71	0.637	1.449	0.739	48
ZINC04349887	0.668	0.668	1.448	0.78	49
ZINC08873138	0.673	0.599	1.448	0.775	50

References

(1) Wetzel, S.; Klein, K.; Renner, S.; Rauh, D.; Oprea, T. I.; Mutzel, P.; Waldmann, H. Interactive Exploration of Chemical Space with Scaffold Hunter. *Nat. Chem. Biol.* **2009**, *5* (8), 581–583.

(2) Muchmore, S. W.; Souers, A. J.; Akritopoulou-Zanze, I. The Use of Three-Dimensional Shape and Electrostatic Similarity Searching in the Identification of a Melanin-Concentrating Hormone Receptor 1 Antagonist. *Chem. Biol. Drug Des.* **2006**, *67* (2), 174–176.
